# Supplementary material for: Estimating disorder probability based on polygenic prediction using the BPC approach
Source: Nat Commun. 2025 Sep 26;16:8443. doi: 10.1038/s41467-025-62929-x (PMC12475198; doi:10.1038/s41467-025-62929-x)
Supplement: Supplementary file 3 — Reporting Summary [file 41467_2025_62929_MOESM3_ESM.pdf]

nature portfolio

Corresponding author(s): Emil Uffelmann

Last updated by author(s): Jun 26, 2025

Reporting Summary

Nature Portfolio wishes to improve the reproducibility of the work that we publish. This form provides structure for consistency and transparency in reporting. For further information on Nature Portfolio policies, see our Editorial Policies and the Editorial Policy Checklist.

Statistics

For all statistical analyses, confirm that the following items are present in the figure legend, table legend, main text, or Methods section.

n/a	Confirmed	
☐	☒	The exact sample size (n) for each experimental group/condition, given as a discrete number and unit of measurement
☒	☐	A statement on whether measurements were taken from distinct samples or whether the same sample was measured repeatedly
☒	☐	The statistical test(s) used AND whether they are one- or two-sided<br>*Only common tests should be described solely by name; describe more complex techniques in the Methods section.*
☐	☒	A description of all covariates tested
☒	☐	A description of any assumptions or corrections, such as tests of normality and adjustment for multiple comparisons
☐	☒	A full description of the statistical parameters including central tendency (e.g. means) or other basic estimates (e.g. regression coefficient) AND variation (e.g. standard deviation) or associated estimates of uncertainty (e.g. confidence intervals)
☒	☐	For null hypothesis testing, the test statistic (e.g. F, t, r) with confidence intervals, effect sizes, degrees of freedom and P value noted<br>*Give P values as exact values whenever suitable.*
☐	☒	For Bayesian analysis, information on the choice of priors and Markov chain Monte Carlo settings
☒	☐	For hierarchical and complex designs, identification of the appropriate level for tests and full reporting of outcomes
☒	☐	Estimates of effect sizes (e.g. Cohen's d, Pearson's r), indicating how they were calculated

Our web collection on statistics for biologists contains articles on many of the points above.

Software and code

Policy information about availability of computer code

Data collection	-
Data analysis	Scripts to apply the BPC approach can be downloaded from https://github.com/euffelmann/bpc.

For manuscripts utilizing custom algorithms or software that are central to the research but not yet described in published literature, software must be made available to editors and reviewers. We strongly encourage code deposition in a community repository (e.g. GitHub). See the Nature Portfolio guidelines for submitting code & software for further information.

Data

Policy information about availability of data

All manuscripts must include a data availability statement. This statement should provide the following information, where applicable:

- Accession codes, unique identifiers, or web links for publicly available datasets
- A description of any restrictions on data availability
- For clinical datasets or third party data, please ensure that the statement adheres to our policy

Individual-level data from the Psychiatric Genomics Consortium (https://pgc.unc.edu/) and the UK Biobank (https://www.ukbiobank.ac.uk/enable-your-research/apply-for-access) cannot be shared freely, but an access application is required first. The GWAS summary statistics used in the UKB analyses can be requested or downloaded from the following web pages: Breast Cancer (https://bcac.ccge.medschl.cam.ac.uk/bcacdata/oncoarray/oncoarray-and-combined-summary-result/gwas-summary-associations-breast-cancer-risk-2020/); BMI (https://portals.broadinstitute.org/collaboration/giant/index.php/GIANT_consortium_data_files);

Coronary Artery Disease (http://www.cardiogramplusc4d.org/data-downloads/#); Inflammatory Bowel Disease (https://www.ibdgenetics.org/); Multiple Sclerosis (https://imsgc.net/?page_id=31); Prostate Cancer (http://practical.icr.ac.uk/blog/?page_id=8164); Rheumatoid Arthritis (https://data.cyverse.org/dav-anon/iplant/home/kazuyoshiishigaki/ra_gwas/ra_gwas-10-28-2021.tar); Type 2 Diabetes (https://diagram-consortium.org/downloads.html). GWAS summary statistics for Major Depression and Schizophrenia can be downloaded from the PGC website (https://pgc.unc.edu/for-researchers/download-results/).
1000 Genomes reference files can be downloaded from https://ctg.cncr.nl/software/magma.

Research involving human participants, their data, or biological material

Policy information about studies with human participants or human data. See also policy information about sex, gender (identity/presentation), and sexual orientation and race, ethnicity and racism.

Reporting on sex and gender	No sex/gender analyses were performed.
Reporting on race, ethnicity, or other socially relevant groupings	Only individuals of European ancestry were considered in this study.
Population characteristics	Population characteristics were not of importance for the current study, as the primary goal was to evaluate and compare methods to predict disorder probabilities and not to define risk in any specific population. Individuals from the PGC datasets did not contain any information on covariates. Individuals from the UKB dataset are a sample from the general population in the United Kingdom; further detailed information can be obtained here https://dx.plos.org/10.1371/journal.pmed.1001779
Recruitment	*Describe how participants were recruited. Outline any potential self-selection bias or other biases that may be present and how these are likely to impact results.*
Ethics oversight	*Identify the organization(s) that approved the study protocol.*

Note that full information on the approval of the study protocol must also be provided in the manuscript.

Field-specific reporting

Please select the one below that is the best fit for your research. If you are not sure, read the appropriate sections before making your selection.

☒ Life sciences ☐ Behavioural & social sciences ☐ Ecological, evolutionary & environmental sciences

For a reference copy of the document with all sections, see nature.com/documents/nr-reporting-summary-flat.pdf

Life sciences study design

All studies must disclose on these points even when the disclosure is negative.

Sample size	Samples sizes are provided in the paper. The power of Polygenic Score analyses depends mostly on the size of the GWAS training sample. We have selected the largest publicly available GWASs for our analyses. The sample size of testing samples was based on availability, where we included either all available cases or selected a max total sample size of 20,000 individuals.
Data exclusions	-
Replication	The aim was to evaluate a new method, which was "replicated" across several disorders.
Randomization	-
Blinding	-

Reporting for specific materials, systems and methods

We require information from authors about some types of materials, experimental systems and methods used in many studies. Here, indicate whether each material, system or method listed is relevant to your study. If you are not sure if a list item applies to your research, read the appropriate section before selecting a response.

Materials & experimental systems

n/a	Involved in the study
☒	☐ Antibodies
☒	☐ Eukaryotic cell lines
☒	☐ Palaeontology and archaeology
☒	☐ Animals and other organisms
☒	☐ Clinical data
☒	☐ Dual use research of concern
☒	☐ Plants

Methods

n/a	Involved in the study
☒	☐ ChIP-seq
☒	☐ Flow cytometry
☒	☐ MRI-based neuroimaging

Plants

Seed stocks	-
Novel plant genotypes	-
Authentication	-
